# Probiotics combined with aminosalicylic acid affiliates remission of ulcerative colitis: a meta-analysis of randomized controlled trial

**DOI:** 10.1042/BSR20180943

**Published:** 2019-01-18

**Authors:** Lijun Peng, Yan Zhong, Aiping Wang, Zhisheng Jiang

**Affiliations:** 1 Medical Record Statistics Office and Library, The Pediatric Academy of University of South China (Hunan Children’s Hospital), Changsha, Hunan Province 410007, People’s Republic of China; 2 Health Care Institute of Hunan Children’s Hospital, Changsha, Hunan Province 410007, People’s Republic of China; 3 Institute of Clinical Medicine, Nanhua Affiliated Hospital, University of South China, Hengyang, Hunan Province 421001, People’s Republic of China; 4 Institute of Cardiovascular Disease and Key Lab for Arteriosclerology of Hunan Province, University of South China, Hengyang, Hunan Province 421001, People’s Republic of China

**Keywords:** mesalazine, probiotics, sulfasalazine, ulcerative colitis

## Abstract

We conducted a meta-analysis to evaluate the effect of probiotic combined with aminosalicylic on induction remission maintenance treatment of ulcerative colitis (UC). We conducted systematic searches in several Chinese and English databases from inception to June 2018, screening randomized controlled trials about effect of probiotics combined with aminosalicylic acid on UC. The evaluation indicator was the rate of remission. The relative risk (RR) and 95% confidence interval (CI) were calculated. A total of 27 studies with 1942 patients were included. The results indicated that the remission rate was significantly higher in the group using probiotics combined with aminosalicylic acid than that in the group using aminosalicylic acid alone (RR = 1.40, 95% CI: 1.27–1.53, *P*=0.000). The subgroup analysis indicated that probiotics combined with aminosalicylic acid can significantly elevate the remission rate in both mild to moderate (RR = 1.33, 95% CI: 1.16–1.54, *P*=0.000) and active stage (RR = 1.40, 95% CI: 1.27–1.64, *P*=0.000) UC. In different number of bacterium, drug types and treatment periods, the combination with probiotics can significantly increase the remission rate UC. The funnel plot shows slight publication bias. Probiotics in conjunction with aminosalicylic can obviously increase the clinical remission rate of activity UC than drug alone. There was no significant difference between combined with mesalazine group and salicylazosulfapyridine group.

## Introduction

Ulcerative colitis (UC) is a chronic intestinal inflammatory disease with increasing prevalence worldwide [1]. Chronic inflammation and colitis in colonic mucosa are the main features of UC. The disease course of UC could be very long and usually accompanied by persistent symptoms. It is quite difficult for UC to be cured [2]. UC affects the life quality of patients. Patients who have longer disease course of UC are more likely to get colon cancer [3]. The etiology and pathogenesis of UC have been extensively studied. Various hypotheses were proposed by different researchers. But until now, none of those hypotheses are perfect. At present, scholars believe that genetic factors, immune factors, intestinal mucosa barrier function factors, environmental factors, and microorganism factors might be related to UC [4]. Since UC is considered as an autoimmune disease, immune factors seem to play an important role in UC [5].

The treatment of UC is mainly anti-inflammation and immune response regulation [6]. In recent years, studies have found that severe intestinal flora imbalances are closely related to the occurrence of UC, so the research and application of microecological agents are getting more and more attention [7–9]. Accumulating evidences have suggested that intestinal microbiota plays an important role in the pathogenesis of this disorder [10,11]. Therefore, increasing clinical trials have been carried out to explore the effects of different probiotics in treating UC and suggested that probiotics could have beneficial effects on UC patients. However, many of these studies either have relatively small sample size or are not randomized controlled trials (RCTs). The meta-analysis based on a large data of RCTs could make the evidence more convincing. The present paper summarized the available literature before June 2018, which concerned the probiotic intervention studies for the management of UC, comparing the effects between probiotics and placebo, no additive treatment to standard therapy or mesalazine, addressing the clinical outcome parameters in UC subjects.

## Materials and methods

We followed the Cochrane Handbook for Systematic Reviews of Intervention to conduct this meta-analysis [12]. The results were reported in compliance with the Preferred Reporting Items for Systematic Reviews and Meta-Analysis (PRISMA) [13]. The ethical approval is not applicable to the present study.

### Literature search

We conducted systematical online searches in the PubMed, Web of science, Embase, Wanfang, VIP (VIP Database for Chinese Technical Periodicals), and China National Knowledge Infrastructure database from inception to June 10, 2018. The following search words were used: probiotics, UC, RCTs, induction of remission, aminosalicylic acid, mesalazine, and sulfasalazine. We placed language restriction to Chinese and English. We also checked the reference lists of previous reviews to identify potentially eligible studies.

### Criteria for inclusion and exclusion

Two investigators (P.L.J. and Z.Y.) independently carried out the initial search. Any discrepancy was resolved by discussion and consensus. The included study had to meet the following criteria: (1) Population: children or adults with UC regardless of clinical setting. (2) Intervention: probiotics in conjunction with mesalazine or sulfasalazine or aminosalicylic acid. (3) Comparison: mesalazine or sulfasalazine or aminosalyicylic acid alone (4) Primary outcomes: remission rate. The latest data were used for duplicates. The reviews, comments, letters, animal study, or experimental study was excluded. Study with unavailable data were also excluded.

### Data extraction

Two authors independently performed the data extraction using a standard excel sheet. Any disagreements were solved by consensus. For each study, the following information was extracted: the first author, publication year, degree of severity (mild to moderate, active stage), intervention versus comparison, probiotics types and number, doses for probiotics, treatment period (week), trial group (event and non-event), control (event vs non-event) sample size. We also contacted the corresponding authors to obtain the information, if required. We only focused on remission rate of patients with UC in the present study.

### Assessment of quality

We used the Cochrane risk of bias tool to assess the quality of included studies [14]. This evaluation tool consists of random sequence generation, allocation concealment, blinding of participants, personnel-to-study protocol, blinding of outcome assessment, incomplete outcomes data, selective reporting, and other bias. The studies were classified as high risk, low risk, and unclear risk of bias according to the above criteria. The studies with more than one key domains were considered high risk of bias, those without all domains were considered low risk of bias. Otherwise, they were considered to be at unclear risk of bias.

### Statistical analysis

In the present study, the relative risks (RRs) with 95% confidence intervals (CIs) were calculated to quantify the effect of probiotics combined with aminosalicylic acid on UC. Heterogeneity within studies was evaluated using *I^2^* statistic and Chi-square test, the *I*^2^>50% or *P*<0.05 indicated significant heterogeneity [15,16]. The random-effect model was used for heterogeneity. Subgroup analyses were also conducted in the following category: severity (mild to moderate vs active stage), number of probiotics (one type, two types, and three types), type of drug (mesalazine vs salicylazosulfapyridine [SASP]) and treatment period (4, 8, and 12 weeks). Sensitivity analysis was conducted to test the stability of pooled results. Publication bias was assessed by visually inspecting a funnel plot and trim and filled funnel plot, and the Begg and Egger’s test was also used [17]. All statistical analyses were completed using Stata 13.0 and RevMan 5.0. *P*<0.05 was considered significance.

## Results

### Study selection

**Figure 1:**
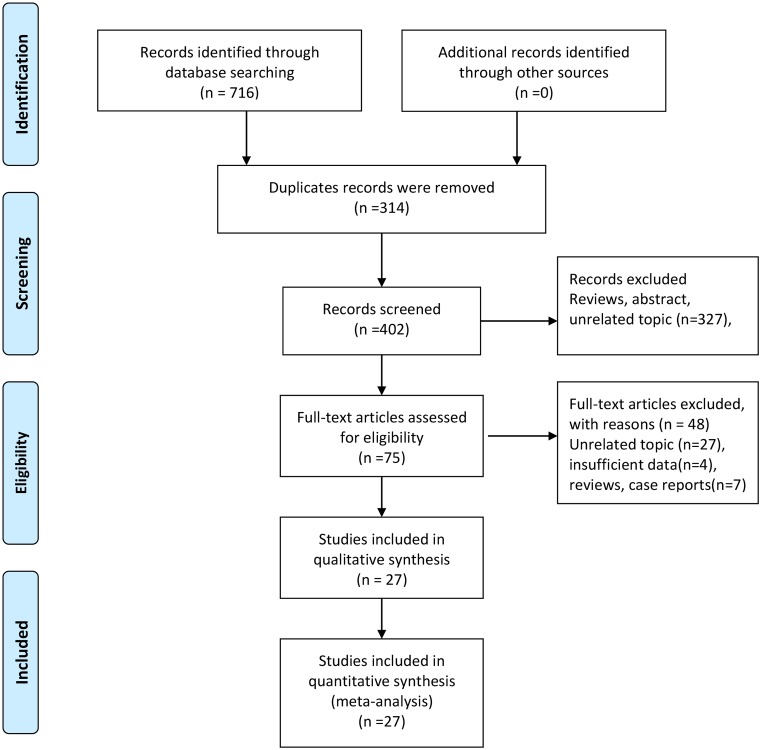
The flow chart of study selection

The flowchart of study selection was presented in Figure 1. We first obtained 716 records from 6 online databases. After 314 duplicates records were removed, 402 records were prepared for further screening. 327 records were excluded by scanning titles and abstracts. We got 75 records for full-text articles assessed for eligibility. 49 studies were excluded including 28 unrelated topics, 4 studies with insufficient data, 7 reviews, and case reports. Twenty-seven studies were finally included in the qualitative and quantitative synthesis (Supplementary File S1).

### General characteristics of included studies

The general characteristics of included studies were presented in Table 1. These studies were published from 2007 to 2018. The sample sizes ranged from 34 to 129, with a total of 1942 patients. Among these studies, 10 studies were conducted among patients with active stage UC, and 16 studies were conducted in patients with mild-to-moderate UC. For control group, 4 studies used SASP only, and 23 studies used mesalazine only. Twenty studies involved seven types of bacterium, including *Bifidobacterium, Lactobacillus, Streptococcus thermophilus, Enterococcus faecalis, Clostridium butyricum, Bacillus subtilis*, and *Saccharum*. Nine studies had one type of bacterium, two studies had two types of bacterium, and fifteen studies included three types of bacterium. The treatment period of most studies were 8 weeks, and eight studies were 4 weeks, and three studies were 12 weeks. The remission rate of trial group ranged from 15.4 to 82.9%, and the control group ranged from 7.7 to 71.9%.

**Table 1:** Characteristics of included study in the meta-analysis

Author	Year	Degree of severity	Control group	Probiotics	Dose/d	Treatment period (weeks)	Trial (E/N)		Control (E/N)		Sample size
Cui [37]	2007	Mild to moderate	Mesalazine	1,2,3	1.5 × 10^7^	12	41	24	46	18	129
Fan [38]	2013	Active stage	Mesalazine	1,2,4	6 × 10^7^	8	15	10	8	17	50
Feng [39]	2012	Mild to moderate	Mesalazine	2	2 × 10^11^	8	11	19	9	21	60
Fu [40]	2012	Mild to moderate	Mesalazine	1,2,4	6 × 10^7^	8	19	16	14	21	70
Gao [41]	2013	Mild to moderate	Mesalazine	1,2,4	6 × 10^7^	4	21	29	21	29	100
Huang [42]	2012	Mild to moderate	SASP	1,2,4	6 × 10^7^	12	19	12	12	19	62
Liu [43]	2007	Mild to moderate	SASP	2	4.2 × 10^2^	4	34	7	23	17	81
Liu [44]	2010	Mild to moderate	Mesalazine	1,2,4	6 × 10^7^	4	17	13	12	16	58
Luo [45]	2008	Mild to moderate	Mesalazine	1,2,4	6 × 10^7^	8	13	12	9	19	53
Tu [53]	2011	Mild to moderate	Mesalazine	1,2,4	6 × 10^7^	4	15	23	10	28	76
Wang [46]	2013	Mild to moderate	Mesalazine	1,2,3	3 × 10^7^	4	23	10	16	14	63
Wang [47]	2013	Mild to moderate	Mesalazine	1,2,4	6 × 10^7^	8	25	10	19	16	70
Yu [48]	2012	Active stage	Mesalazine	1,2,4	6 × 10^7^	8	16	15	10	21	62
Yuan [49]	2012	Active stage	Mesalazine	1,2,4	6 × 10^7^	4	12	6	7	11	36
Zhang [50]	2013	Mild to moderate	Mesalazine	124	8 × 10^7^	8	16	10	8	14	48
Zhang [51]	2012	Active stage	Mesalazine	5	3.78 × 10^7^	8	26	21	18	29	94
Zhou [52]	2009	Mild to moderate	Mesalazine	1,2,4	6 × 10^7^	4	9	11	5	9	34
Chen [27]	2017	Active stage	Mesalazine	6	–	8	8	26	4	30	68
Liang [28]	2017	Active stage	Mesalazine	6	0.5 g, TID	8	24	26	19	31	100
Xu [29]	2016	Active stage	Mesalazine	7	0.5 g, BID	8	16	15	11	20	62
Peng [30]	2017	Active stage	Mesalazine	4	0.5 g, TID	8	35	28	24	39	126
Chen [31]	2015	Active stage	SASP	4,6	0.5 g, TID	8	4	22	2	24	52
Gong [32]	2015	Active stage	Mesalazine	1	0.5 g, TID	8	28	12	19	21	80
Ou [36]	2014	Mild to moderate	SASP	2	0.5 g, TID	12	28	26	17	37	108
Wang [35]	2014	Mild to moderate	Mesalazine	1,2,4	0.5 g, TID	8	24	16	14	26	80
Hua [34]	2015	Mild to moderate	Mesalazine	1,2,3	0.5 g, TID	4	21	11	13	19	64
Zhang [33]	2015	Mild to moderate	Mesalazine	1,2,3	0.5 g, TID	8	15	15	6	20	56

*E, event; N, non-event; 1, Bifidobacterium; 2, Lactobacillus; 3, Streptococcus thermophilus; 4, Enterococcus faecalis; 5, Clostridium butyricum; 6, Bacillus subtilis; 7, Saccharum. TID, three times per day; BID, twice per day.

### Assessment of quality

The Supplementary Figures S2 and S3 presented the details of risk of bias. Overall, 12 studies were categorized as being at high risk, and the rest of studies were unclear risk of bias. The primary reason is the blinding of participants and personnel (performance bias). These studies did not report whether blinding method was used. The random sequence generation was perfect in all studies.

### Pooled results

**Figure 2:**
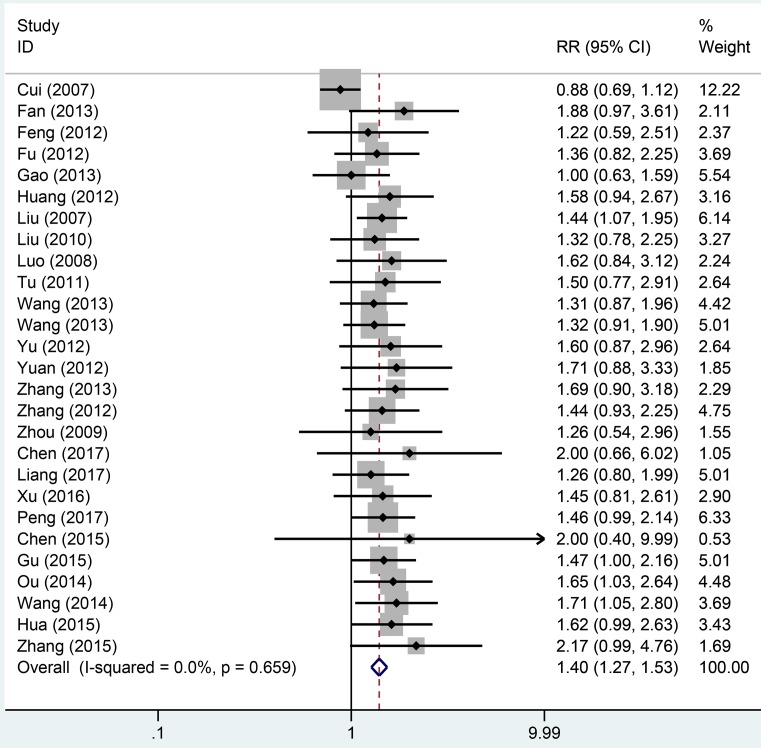
Forest plot of effect of probiotics combined with aminosalicylic acid on UC

**Figure 3:**
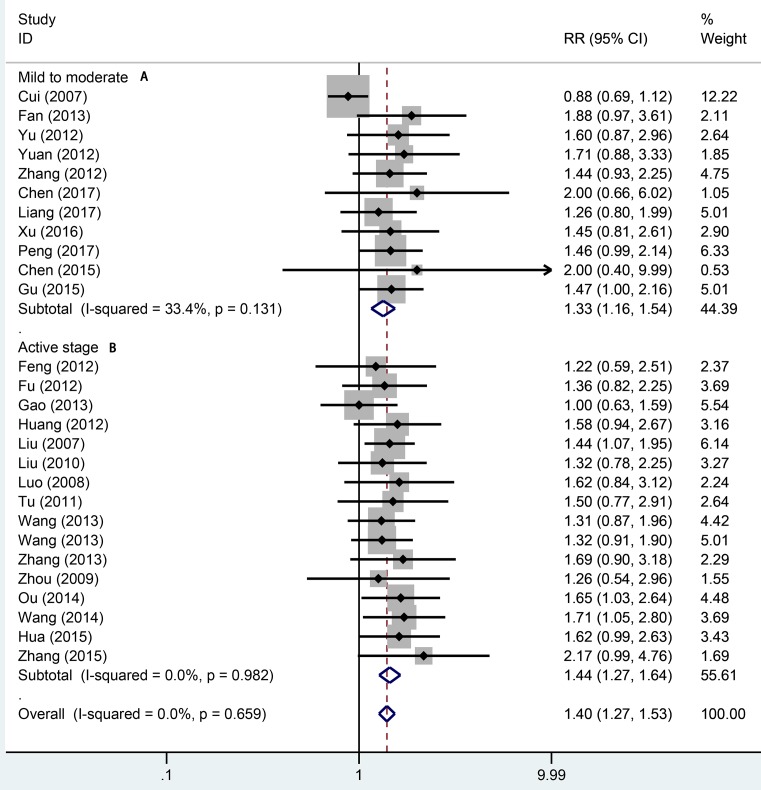
Forest plot of effect of probiotics combined with aminosalicylic acid on different stage of UC A, mild to moderate; B, active stage.

**Figure 4:**
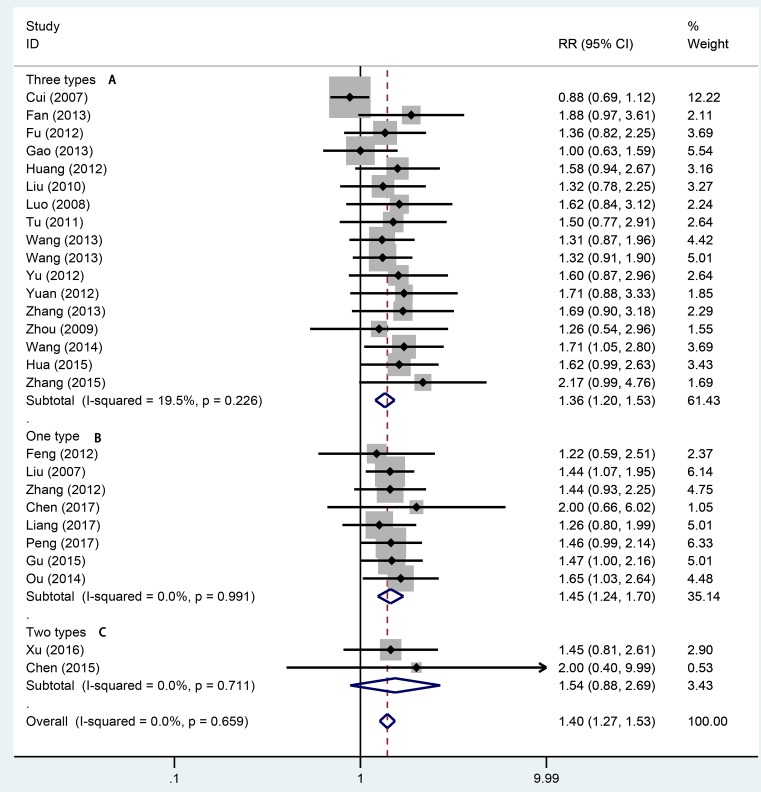
Forest plot of effect of probiotics combined with aminosalicylic acid on UC in different number of protiotics A: three types; B: one type; C: two types.

**Figure 5:**
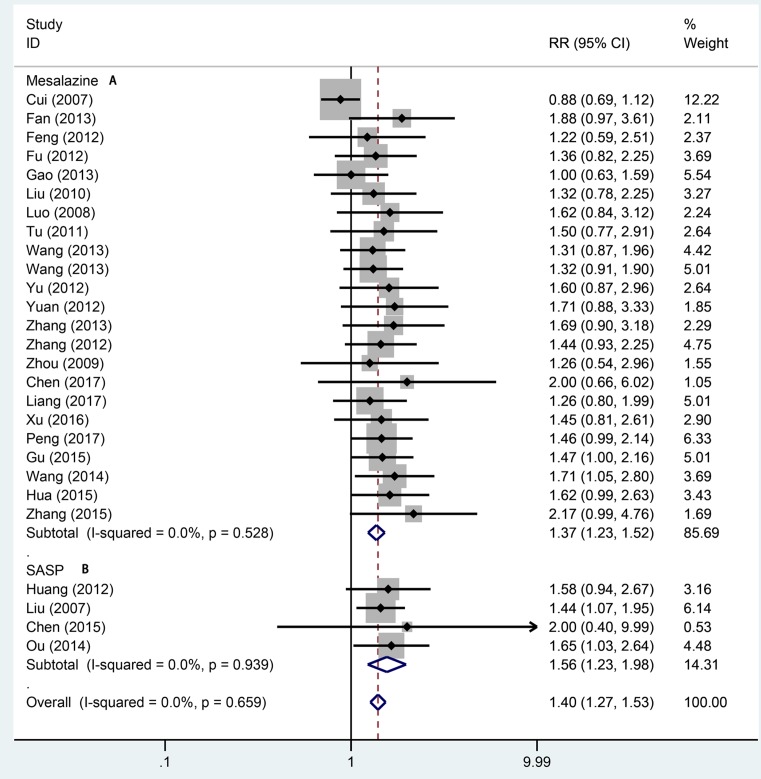
Forest plot of effect of probiotics combined with different aminosalicylic acid on UC A: mesalazine; B: SASP.

**Figure 6:**
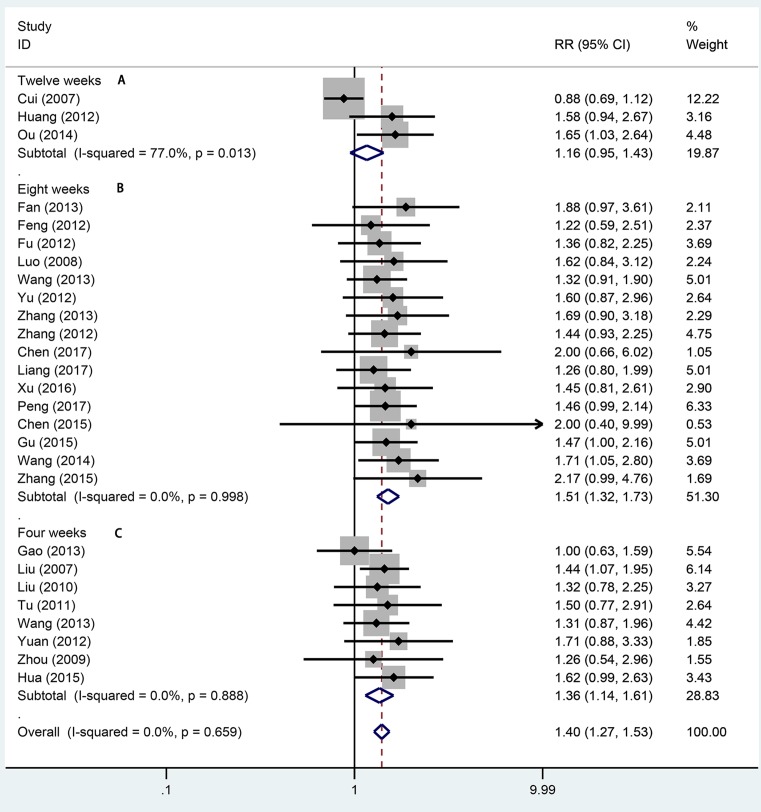
Forest plot of effect of probiotics combined with aminosalicylic acid on UC in different treatment period A: 12 weeks; B: 8 weeks; C: 4 weeks.

Twenty-seven studies totaling 1942 provided data on remission rate. Compared with aminosalicylic acid alone, the combination with probiotics significantly increased remission rate (RR = 1.40, 95% CI: 1.27–1.53, *P*=0.000, Figure 2) without heterogeneity (*I*^2^=0.0%, *P*=0.659). The subgroup analysis indicated that probiotics combined with aminosalicylic acid can significantly elevate the remission rate in both mild to moderate (RR = 1.33, 95% CI: 1.16–1.54, *P*=0.000, Figure 3A) and active stage (RR = 1.40, 95% CI: 1.27–1.64, *P*=0.000, Figure 3B) UC. One and three types showed that combination with probiotics can affiliate the remission of UC (RR = 1.36, 95% CI: 1.20–1.53, *P*=0.000; RR = 1.45, 95% CI: 1.24–1.70, *P*=0.000, Figure 4). There was no significance for two types of bacterium between the trial and control group (RR = 1.54, 95% CI: 0.88–2.69, *P*=0.130, Figure 4). For different mesalazine or SASP, the combination was still better than drug only. Significant differences were found in both types of drug (Figure 5, RR = 1.37, 95% CI: 1.23–1.52, *P*=0.000 vs RR = 1.56, 95% CI: 1.23–1.98, *P*=0.000). We also conducted subgroup analysis in different treatment periods, the results from fixed-effect model indicated that significant remission rate differences were found (8 weeks: RR = 1.51, 95% CI: 1.32–1.73, *P*=0.000; 4 weeks: RR = 1.36, 95% CI: 1.14–1.61, *P*=0.001, Figure 6). For 12 weeks, there were no significant differences (RR = 1.16, 95% CI: 0.95–1.43, Figure 6).

### Sensitivity analysis

The sensitivity analysis was conducted by excluding one study each time and pooled the rest studies. The results indicated that no significant alteration was found (Supplementary Figure S4), which showed that the pooled results were stable.

### Publication bias

**Figure 7:**
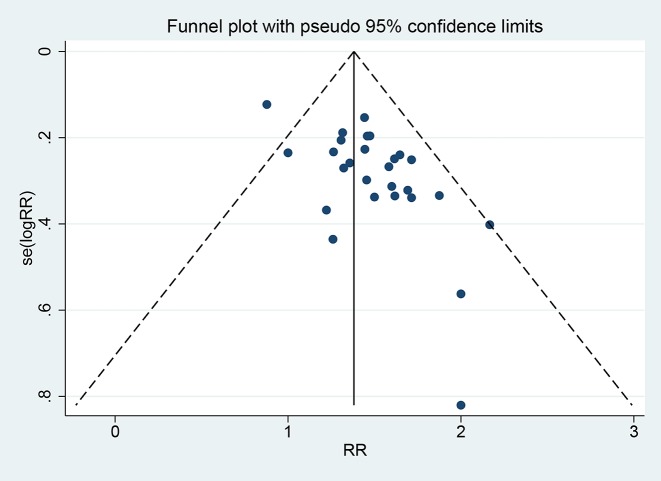
Funnel plot of publication bias

The funnel plot was presented in Figure 7. There was a slight asymmetry in the funnel plot, which means publication bias may exists. The Begg and Egger’s test indicated that some publication bias existed (*Z*=2.000, *P*=0.045, *t*=7.050, *P*=0.000). We also used the trim and filled funnel plot to assess the publication bias. Eleven studies were needed to make the funnel plot balance (Supplementary Figure S5).

## Discussion

The present study comprehensively and systematically reviewed the current available literature and found that: (1) probiotics in conjunction with aminosalicylic acid can significantly increase the remission rate of UC compared with aminosalicylic acid alone. There was no significant difference between combined with mesalazine group and SASP group. (2) The results remained consistent in different number of bacterium, severity of UC, type of combined drug, and treatment periods (4 vs 8 weeks). (3) For a 12-week treatment, no significant effect difference was found between drug combination and single drug.

The pathological and physiological mechanism of UC was complicated, including at least four interacting factors: gene, immune system, intestinal flora, and environment [18]. Compared with normal healthy people, the intestinal flora of most patients with UC had changed. It was reported that the intestinal flora had changed regardless of active period or the relieved period for patients with UC [19]. The number of *Pseudomonas, Bifidobacterium, Helicobacter spiralis, Fragile pseudomonas*, and *Clostridium difficile* had decreased while the number of Enterococcus and Bacillus increased. Therefore, it is theoretically possible to alleviate the active period of UC by correcting the imbalance of bacterial flora through probiotics [20]. The possible mechanisms of probiotics in the treatment of UC included inhibiting the growth pathogenic bacteria, improving the function of epithelial mucosal barrier, regulating immunity, and reducing the secretion of proinflammatory factors. Previous studies evaluated the maintenance effect of probiotics versus that of aminosalicyates on UC. A meta-analysis with six RCTs and a total of 721 participants indicated that no significant difference was observed between probiotics and aminosalicylate (RR = 1.08, 95% CI: 0.91–1.28). However, the number of this meta-analysis was limited [21]. Sang et al. [22] evaluated the induction of remission and maintenance effect of probiotics of UC. Compared with the non-probiotics group, the remission rate for UC patients who received probiotics was not significantly altered (RR = 1.35, 95% CI: 0.98–1.85). Nevertheless, the remission rate of UC which received probiotics was increased (RR = 2.00, 95% CI: 1.35–2.96). Another study with 23 RCTs and a total of 1763 patients evaluated whether probiotics were beneficial at all stages of the treatment in inflammatory bowel disease or superior to placebo. They found that probiotics can significantly increase the remission rate in patients with active UC (*P*=0.010) compared with placebo group. Unfortunately, similar effect on maintaining remission rate of UC was found between probiotics and 5-aminosalicylic acid [23]. These studies suggested that probiotic treatment was more effective than placebo in maintaining remission of UC and was equivalent compared with aminosalicylic acid.

In the past several years, more and more researchers have focused on combined drugs.

Previous studies indicated that the maintaining effect of probiotics for active stage UC was unclear, and the induction remission of aminosalicylic acid was obvious [24]. Whether patients with UC can benefit a lot from combination of probiotics with aminosalicylic acid still remains unclear. Currently, the aminosalicylic acid agents are commonly used in the treatment of UC, including SASP and mesalazine. Patients with UC tended to use large amounts of antibiotics for a long time [25]. The number of normal bacteria in the intestinal tract is reduced. The SASP was decomposed into 5-aminosalicylic acid by bacteria. The effect of aminosalicylic acid greatly decreased. At the same time, SASP itself has a certain bacteriostatic effect, and also inhibited the intestinal bacteria decomposition of SASP [26]. Both of the two reasons lowered the concentration of 5-aminosalicylic acid, which makes the therapy effect not ideal. Therefore, if probiotics was used when aminosalicylic acid was taken, this plan can regulate the intestinal flora and increase the concentration of 5-aminopylic acid and improve the curative effect. The present study confirmed this theory and gave a stronger evidence.

The main strengthen of the present study is that we did not find any significant heterogeneity within studies. The present study still has several limitations. First, the quality of included study was moderate, and the primary reason was the lack of blinding methods. Second, a number of included studies are few in some subgroup settings, and more rigorous and well-designed RCTs are needed to confirm our results. Third, most of the studies are from Asian populations that may cause publication bias. In fact, the funnel plot, Begg and Egger’s test also indicated that there was a slight publication bias, which may influence the stability of pooled results. Finally, although we carefully selected published studies, some unpublished data and grey literature were not included, which may influence the estimation.

In conclusion, our meta-analysis suggested that probiotics in conjunction with aminosalicylic can obviously increase the clinical remission rate of activity UC than drug alone. This effect was not influenced by probiotic types, severity of disease, and type of drugs. After a 12-week treatment, no significant difference was observed between combined and single drugs. This result from subgroup analysis should be cautious because of the limited number of studies.

## Supporting information

**Supplementary Figures:** 

## Abbreviations

CI: confidence interval
PRISMA: preferred reporting items for systematic reviews and meta-analysis
RCT: randomized controlled trial
RR: relative risk
SASP: salicylazosulfapyridine
UC: ulcerative colitis
