# Supplementary material for: Probiotics combined with aminosalicylic acid affiliates remission of ulcerative colitis: a meta-analysis of randomized controlled trial
Source: Biosci Rep. 2019 Jan 18;39(1):BSR20180943. doi: 10.1042/BSR20180943 (PMC6340951; doi:10.1042/BSR20180943)
Supplement: Supplementary file 1 [file bsr20180943_Supp1.pdf]

Supplementary material 1: Lists of included studies in the meta-analysis

1.Xuan-xuan C, Yong Z, Xiang-nong X. Efficacy and safety of probiotics combined with mesalazine in the treatment of ulcerative colitis. J Hunan Normal Univ (Med Sci). 2017; 14:43-45.

2.Liang JS. The efficacy of probiotics adjuvant therapy on IL-23/IL-17 inflammatory axis in patients with ulcerative colitis. Lab Med Clin. 2017; 14:1434-1435, 1438.

3.Xu YC, Feng QQ, Li CA, Xin J. Mesalazine Combined with Probiotics for Ulcerative Colitis. J Nanchang Univ (Med Sci). 2016; 56:47-49.

4.Peng Q. Clinical value of mesalazine Combined with Probiotics for Ulcerative Colitis. Haixia Med. 2017; 29:160-161.

5.Chen LH, Chen ML, Wu FB, W WJ, Xie NN. Clinical efficacy of probiotics combined with sulfasalazine in the treatment of ulcerative colitis. Hainan Med. 2015; 26:970-972.

6.Gong YY, Wang YL, Sun Y. Effect of probiotics on mild to moderate ulcerative colitis during active stage. Chin Health Nutr. 2015; 9:102-103.

7.Zhang YF, Zhang M, Cui SS, Zhang RJ. Probiotics in treatment of mild - moderate ulcerative colitis efficacy. Taishan Med Univ. 2015; 36:604-607.

8.Hua HW. Clinical Observation of Mesalazine Combined with Probiotics in Treatment of Ulcerative Colitis. Guangdong Trace Elem Sci. 2015; 22:47-50.

9.Wang YZ. Analysis on the role of probiotics in ulcerative colitis. Heilongjiang Med. 2014; 38:559.

10. Ou XE, Wan CR. Clinical analysis of selecting probiotics in treatment of

ulcerative colitis. J Qiqihar Univ Med. 2014; 35:2986-2987.

11. Cui J, Xu G, Liu ZJ. Effect if oribuitucs drug treatment of mild and moderate ulcerative colitis. Med Froum. 2007; 28:52-54.

12. Fan WH. The effect of combined therapy of mesalazine and probiotics in the treat!
ment of ulcerative colitis. Med Clin Prac. 2013:75-76.

13. Feng YM, Yu GH, Cheng SW, Yu J, Wang Z. Effect of probiotics on the curative effect of ulcerative colitis. Clini Lab Med. 2012; 11:1527-1528.

14. Fu XJ. Clinical efficacy of combinging probiotic with mesalazine in the treatment of ulcerative colitis. China Health Industry. 2012; 12:17.

15. Gao S. Comparative observation on the clinical efficacy of ulcerative colitis. Chin Foreign Med Research. 2013; 11:29-30.

16. Huang L. Curative effect of the combined treatment with probiotics and sulfasalazine for ulcerative colitis. Sichuan Med. 2012; 33:766-768.

17. Jian-sheng L, Yi T, Xiao-hong Z, Jin L, Ming MA, Yao-zong Y, Jian L. The levels of nitric oxide and superoxide dismutase in serum and intestine mucosal of ulcerative colitis and the influencing of probiotics agents to them. Chin J Postgrad Med. 2007; 30:20-22.

18. Liu Y, Tan RM. Clinical observation of probiotics combined with mesalazine in the treatment of ulcerative colitis. Jilin Med. 2010; 21:2228-2230.

19. Luo Y. Study on the clinical effects of mesalazine combined with probiotics in patients with ulcerative colitis: Shandong Univ; 2008.

20. Wang HY. Efficacy of mesalazine combined with probiotics in the treatment of mild to moderate ulcerative colitis. Shanxi Med. 2013; 42:921-922.

21. Wang R, Xu HM, Zhang BH. The combined application of methalazine and intestinal probiotics in the treatment of ulcerative colitis. J Qiqihar Univ Med. 2013; 34:2547-2548.

22. Yu F, Xu B. Analysis on the effect of mesalazine combined with probiotics in the treatment of ulcerative colitis. Seek Med&Ask Med. 2012; 10:189.

23. Yuan H. Efficacy of probiotics combined with misalazine in the treatment of ulcerative colitis. Chin Med Guide. 2012; 5:483-484.

24. Zhang HM. Efficacy and mechanism of probiotics in the treatment of ulcerative colitis. Chin Modern Doct. 2013; 51:139-141.

25. Zhang ZH. Clinical observation on the treatment of ulcerative colitis with clostridium casein capsule combined with methadine. J Changzhi Med College. 2012; 26:107-108.

26. Zhou XJ. The therapeutic effect of probiotics combined with mesalazine on ulcerative colitis was observed. J Jiangsu Univ. 2009; 19:354-356.

27. Tu ZY, Li YM, Xu W. Mesalazine combined with probiotic for ulcerative colitis：An effect observation of 38 cases. Chin Modern Doct. 2011; 49:91-92.

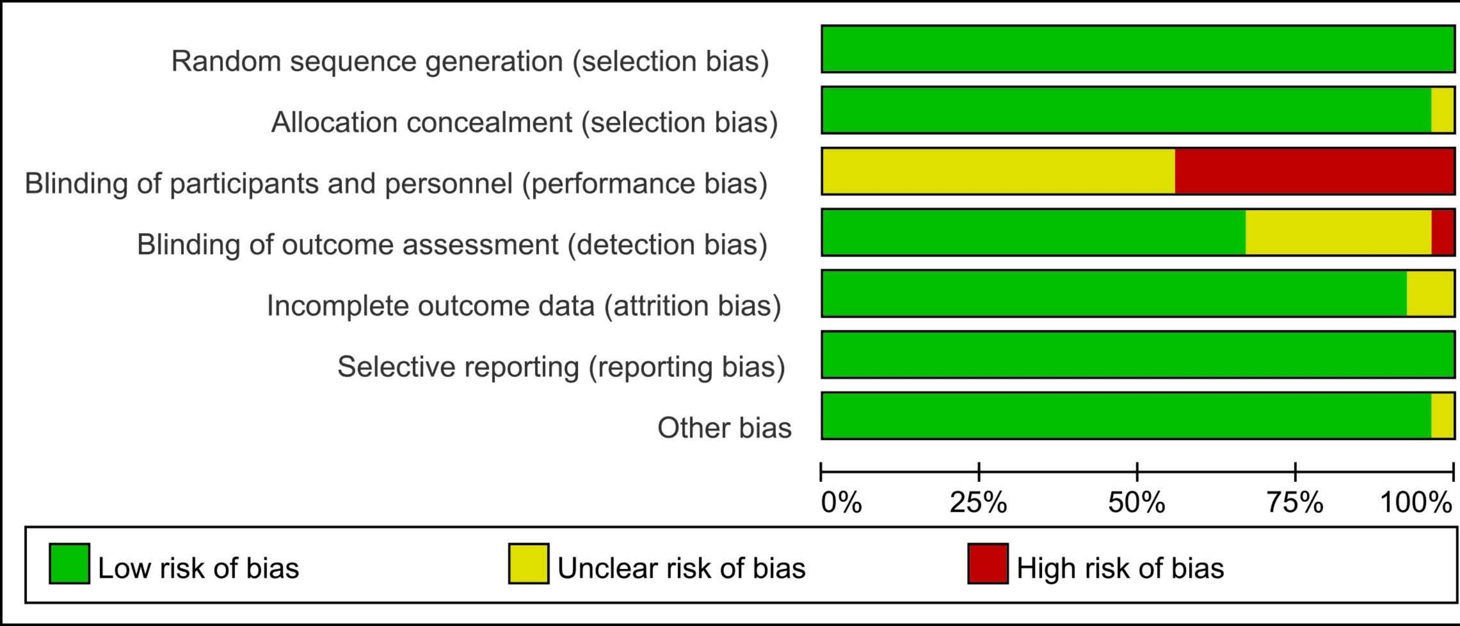
Random sequence generation (selection bias)
Allocation concealment (selection bias)
Blinding of participants and personnel (performance bias)
Blinding of outcome assessment (detection bias)
Incomplete outcome data (attrition bias)
Selective reporting (reporting bias)
Other bias
0%
25%
50%
75%
100%
Low risk of bias
Unclear risk of bias
High risk of bias

	Random sequence generation (selection bias)	Allocation concealment (selection bias)	Blinding of participants and personnel (performance bias)	Blinding of outcome assessment (detection bias)	Incomplete outcome data (attrition bias)	Selective reporting (reporting bias)	Other bias
Chen 2015	+	+	?	+	+	+	+
Chen 2017	+	+	?	+	+	+	+
Cui 2007	+	+	−	?	+	+	+
Fan 2013	+	+	?	+	+	+	+
Feng 2012	+	+	?	+	+	+	+
Fu 2012	+	+	−	+	+	+	+
Gao 2013	+	+	−	?	+	+	+
Gong 2015	+	+	?	+	+	+	+
Hua 2015	+	+	?	+	+	+	+
Huang 2012	+	+	−	+	+	+	+
Liang 2017	+	+	?	?	+	+	+
Liu 2007	+	+	?	?	+	+	+
Liu 2010	+	+	−	+	+	+	+
Luo 2008	+	+	−	+	+	+	+
Ou 2014	+	+	−	+	+	+	+
Peng 2017	+	+	?	?	+	+	+
Tu 2011	+	+	−	+	+	+	+
Wang 2013	+	+	?	?	+	+	+
Wang 2013a	+	?	−	+	+	+	?
Wang 2014	+	+	−	−	+	+	+
Xu 2016	+	+	?	+	?	+	+
Yu 2012	+	+	?	+	+	+	+
Yuan 2012	+	+	−	?	+	+	+
Zhang 2012	+	+	?	?	+	+	+
Zhang 2013	+	+	?	+	+	+	+
Zhang 2015	+	+	−	+	?	+	+
Zhou 2009	+	+	?	+	+	+	+

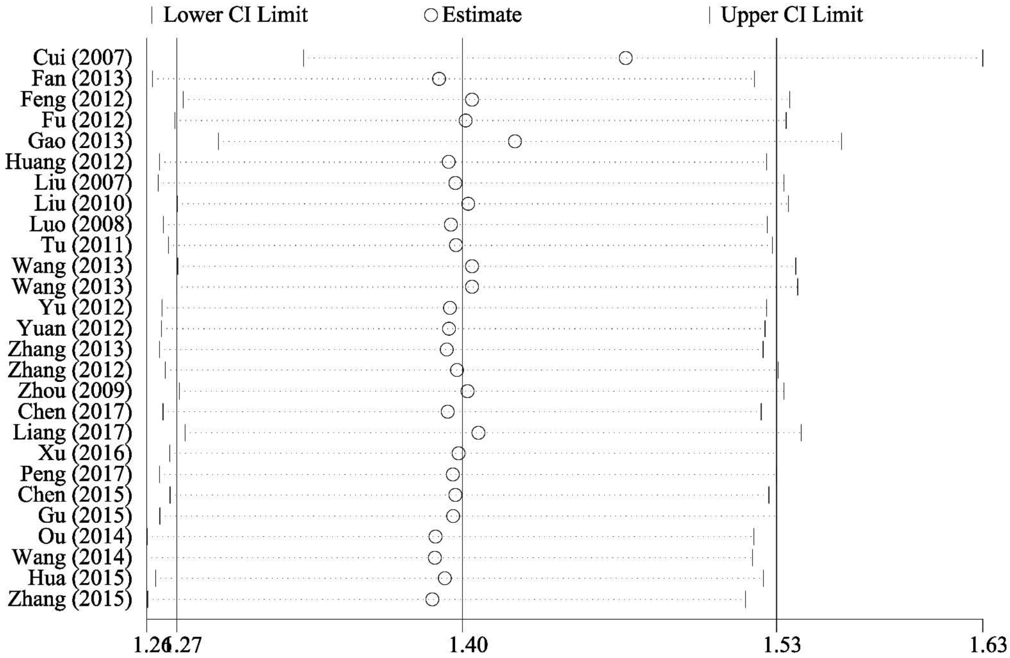

Lower CI Limit
Estimate
Upper CI Limit
Cui (2007)
Fan (2013)
Feng (2012)
Fu (2012)
Gao (2013)
Huang (2012)
Liu (2007)
Liu (2010)
Luo (2008)
Tu (2011)
Wang (2013)
Wang (2013)
Yu (2012)
Yuan (2012)
Zhang (2013)
Zhang (2012)
Zhou (2009)
Chen (2017)
Liang (2017)
Xu (2016)
Peng (2017)
Chen (2015)
Gu (2015)
Ou (2014)
Wang (2014)
Hua (2015)
Zhang (2015)
1.26
1.27
1.40
1.53
1.63

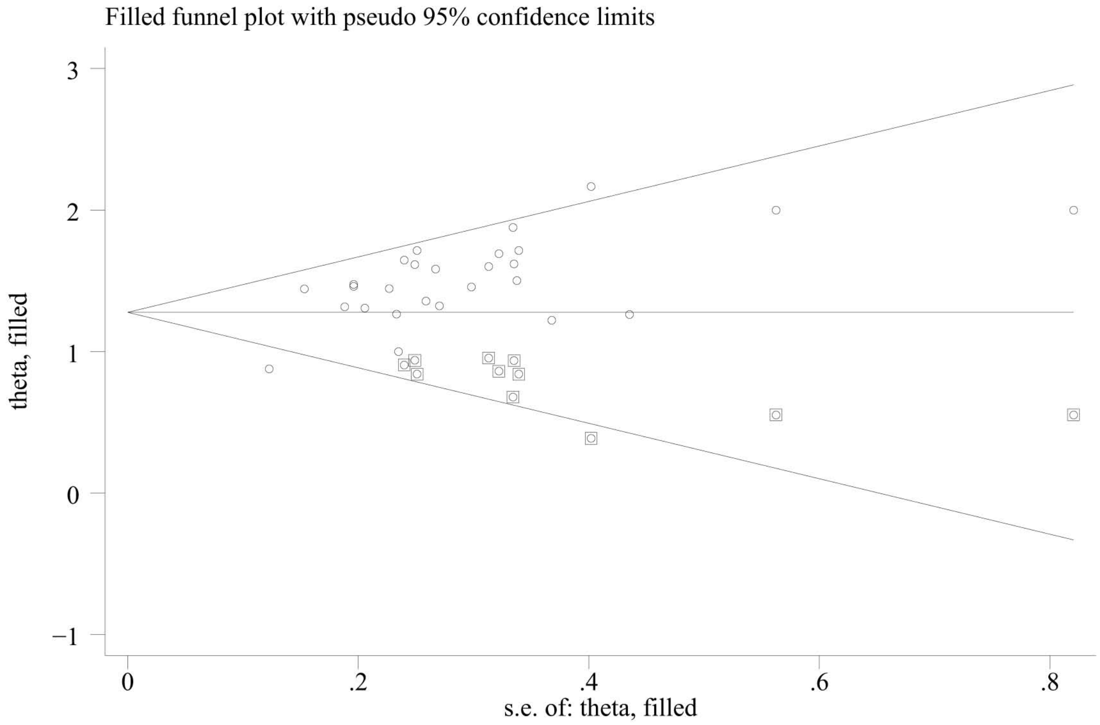
Filled funnel plot with pseudo 95% confidence limits
3
2
1
0
−1
theta, filled
0
.2
.4
.6
.8
s.e. of: theta, filled
